# Late Complications After Aortic Coarctation Repair

**DOI:** 10.3390/jcdd12110450

**Published:** 2025-11-19

**Authors:** Annarita Santoro, Fiorenza De Lisio, Alexandra Fedorovna Bezborodova, Roberto Chiesa, Germano Melissano

**Affiliations:** Division of Vascular Surgery, IRCCS San Raffaele Scientific Institute, Vita-Salute San Raffaele University, 20132 Milan, Italy; santoro.annarita@hsr.it (A.S.); f.delisio@studenti.unisr.it (F.D.L.); a.bezborodova@studenti.unisr.it (A.F.B.); chiesa.roberto@hsr.it (R.C.)

**Keywords:** aortic coarctation, pseudoaneurysm, thoracic aorta, thoracotomy, post coarctation aneurysm, re-coarctation

## Abstract

Aortic coarctation (CoA) is a congenital vascular anomaly characterized by luminal narrowing of the aorta, representing approximately 5–8% of all congenital heart defects, and is frequently associated with a bicuspid aortic valve and additional vascular malformations. The clinical spectrum is broad, ranging from severe neonatal heart failure to asymptomatic systemic hypertension in adulthood, with the severity of presentation directly influencing the timing of diagnosis and therapeutic intervention. Over recent decades, management strategies have transitioned from conventional surgical techniques—such as end-to-end anastomosis, subclavian flap aortoplasty, and patch augmentation—to endovascular modalities including balloon angioplasty and stent implantation, with covered stents now constituting the preferred approach in most cases. Nonetheless, late complications remain clinically significant. Post-coarctation aneurysms (pCoAA), particularly following patch aortoplasty, have been reported in up to 50% of patients and necessitate lifelong imaging surveillance. Re-coarctation persists as a therapeutic challenge, especially in neonates, with recurrence risk influenced by anatomical factors and the initial repair method. Optimal outcomes require an individualized, anatomy-tailored approach that judiciously integrates surgical, endovascular, and hybrid techniques. Lifelong surveillance remains essential to mitigate long-term risks, including systemic hypertension, aneurysm formation, and the need for re-intervention.

## 1. Introduction

**Figure 1:**
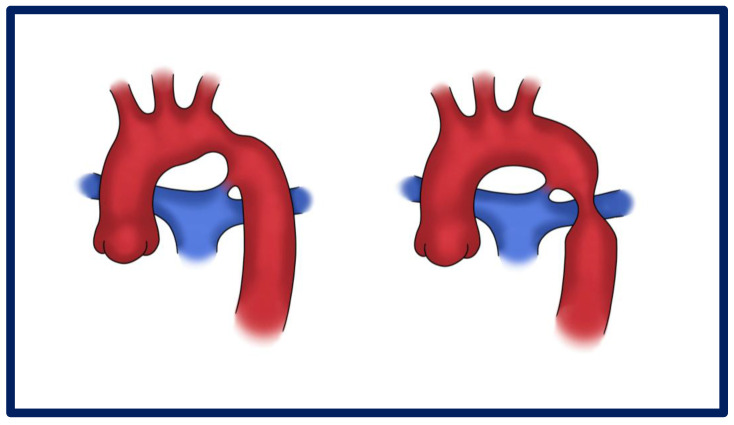
Graphical presentation of different types of CoA—pre-ductal and post-ductal.

**Figure 2:**
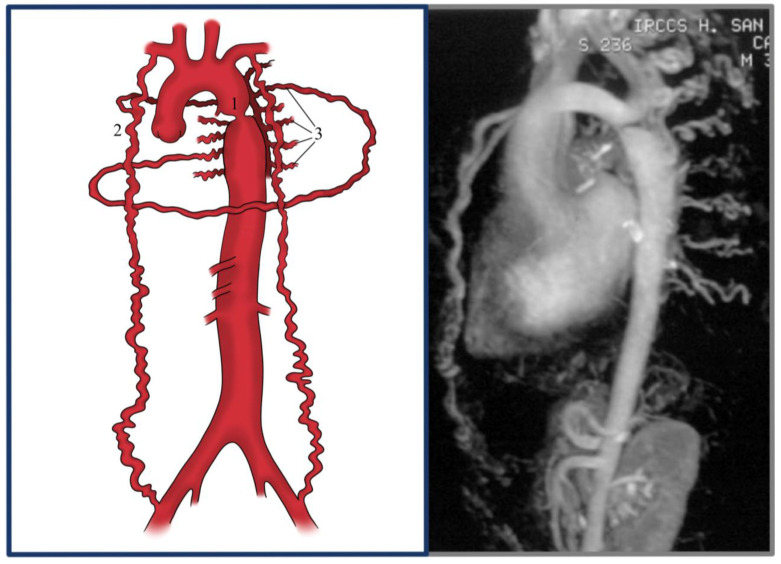
Localized CoA (1) with the internal thoracic artery (2) and intercostal (3) hypertrophy: graphical presentation and 3D MRI.

**Figure 3:**
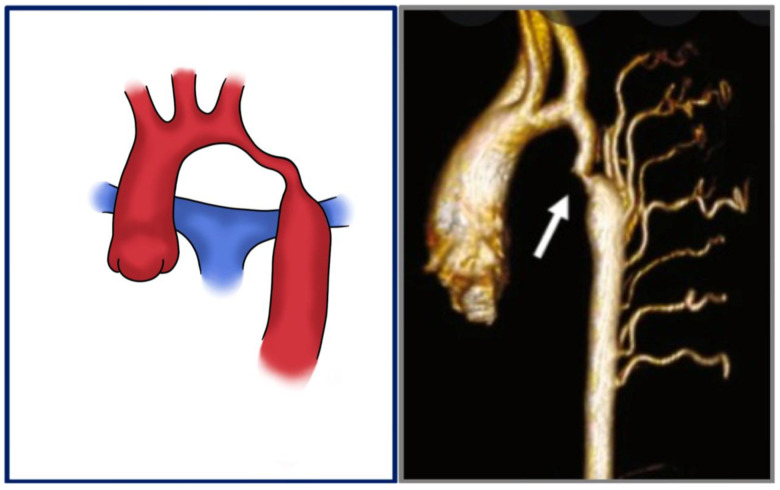
CoA with tubular hypoplasia: graphical presentation and 3D volume rendering. White arrow shows the narrowed aortic segment.

Aortic coarctation (CoA) is a congenital vascular malformation that, although relatively uncommon in the general population, accounts for approximately 5–8% of all congenital heart defects [1]. It is characterized by a narrowing of the aortic lumen (Figure 1), resulting in variable degrees of left ventricular afterload and systemic hypertension [1,2]. The classical form presents as a discrete, localized stenosis typically located at the aortic isthmus—near the site of the ligamentum arteriosus insertion—immediately distal to the origin of the left subclavian artery [3] (Figure 2). Less commonly, the lesion may involve a long-segment hypoplasia of the aortic arch or descending aorta (Figure 3), which may pose greater challenges in both diagnosis and management [1,4,5]. Although the majority of cases are detected in the juxtaductal region, ectopic forms of coarctation have been reported, involving the ascending aorta, thoracic descending aorta, or even the abdominal aorta [6]. Acknowledging these atypical variants is significant, as their presentation may differ significantly and mimic other vascular pathologies [7,8,9].

Coarctation of the aorta can coexist with other cardiovascular anomalies [2]. Notably, anomalies of the right subclavian artery, such as an aberrant origin or retroesophageal course, have been described in association with CoA [10]. Furthermore, there is evidence linking CoA with an increased prevalence of intracranial aneurysms, particularly those involving the circle of Willis [11]. This association points to the role of comprehensive vascular evaluation in affected individuals [11]

One of the most clinically significant associations of CoA is the presence of a bicuspid aortic valve (BAV) [12,13]. This co-occurrence not only reflects a shared embryological origin but also carries important prognostic implications [14]. Several studies showed that patients with both CoA and BAV are at increased risk for progressive aortic dilatation and, subsequently, aortic dissection [15,16].

The clinical presentation of CoA varies significantly depending on the severity of the narrowing. In severe cases, signs such as poor feeding, failure to thrive, differential pulses, or congestive heart failure may emerge within the first weeks of life [17], prompting early diagnosis and timely surgical or catheter-based intervention during infancy [14,18]. In contrast, milder forms may remain asymptomatic or present with nonspecific symptoms such as exertional fatigue or hypertension, leading to delayed diagnosis, often in adolescence or even adulthood [14,19].

This variability in the timing of diagnosis and treatment is a critical factor when evaluating long-term vascular outcomes, particularly the development of post-coarctation aneurysms. Aneurysmal changes in the aortic wall—especially at or near the site of previous repair—can be influenced by both the age at which intervention occurs and the type of procedure performed (e.g., surgical resection with end-to-end anastomosis, patch aortoplasty, or stenting). Patients treated later in life are more likely to have longstanding hypertension and aortic wall stress, which may contribute to unfavorable remodeling and aneurysm formation [20]. Therefore, primary intervention timing and modality must be carefully considered when assessing aneurysm morphology in follow-up imaging, as these factors play a role in risk stratification and surveillance strategies.

## 2. Surgical Approaches for CoA

### 2.1. Open Surgical Repair: Techniques and Considerations

Surgical intervention remains a cornerstone in the management of CoA, particularly in neonates, infants, and complex anatomical presentations [2,17]. Several surgical techniques have been developed over the decades, with the choice of approach guided by patient age, anatomy of the coarctation segment, associated cardiac or vascular anomalies, and institutional experience [21]. The most widely used technique is end-to-end anastomosis, where the narrowed segment of the aorta is excised, and a termino-terminal reconstruction is performed between the two healthy aortic portions, which are then reconnected [2,17]. This approach is typically applied in neonates and infants and is favored for its anatomical simplicity and effectiveness in cases with discrete narrowing [22]. However, in long-segment coarctation or significant arch hypoplasia, this method may not be suitable due to excessive tension on the anastomosis, increasing the risk of re-coarctation [22].

Subclavian flap aortoplasty utilizes the left subclavian artery to surgically enlarge the narrowed aortic segment. This approach is advantageous in small infants, where native tissue may be insufficient for primary repair [22,23,24]. Some papers reported a potential risk of diminished perfusion of the left upper limb or subclavian steal syndrome; however, this evidence is anecdotal with limited clinical significance [24,25].

Patch aortoplasty, in which a synthetic or autologous patch is used to enlarge the narrowed aorta, was once common, especially for long-segment disease. However, this technique has fallen out of favor due to a higher incidence of late aneurysm formation at the repair site [22,26,27].

In selected cases, particularly when associated cardiac defects require correction, median sternotomy and arch reconstruction may be performed. This allows simultaneous treatment of intracardiac anomalies and extensive aortic arch hypoplasia [22,26,27].

The choice of surgical approach must be individualized, taking into account the anatomy of the lesion, the presence of comorbidities, and the long-term risk of complications such as re-coarctation or aneurysm formation. Advances in imaging and surgical techniques continue to improve outcomes, though lifelong follow-up remains essential for all patients.

### 2.2. Endovascular Treatment of Aortic Coarctation: Evolving Role and Techniques

Over the past few decades, endovascular therapy has emerged as a viable and, in many cases, preferred alternative to surgery for the treatment of CoA, particularly in adolescents and adults [28]. This minimally invasive approach offers shorter recovery times, reduced procedural morbidity, and favorable outcomes in selected patients [21,28,29].

Balloon angioplasty was the earliest endovascular technique introduced for CoA repair [28,30]. It involves dilating the narrowed segment of the aorta using an aortic balloon. While effective in relieving obstruction, especially in native coarctation, it carries risks of arterial wall injury, dissection, or aneurysm formation, particularly in older patients or those with calcified or fragile aortas.

To improve outcomes and reduce complications, bare-metal stents were introduced [31]. These provide scaffolding support to maintain luminal patency after balloon dilation, decreasing the risk of restenosis and offering more durable results compared to angioplasty alone [17,28,30].

Nowadays, when endovascular treatment using stenting is planned for CoA repair, covered stents have become the preferred option. These stents combine structural support with a synthetic covering, providing both mechanical relief of stenosis and protection against rupture or dissection, making them the optimal choice in high-risk anatomy [21,28,30].

**Figure 4:**
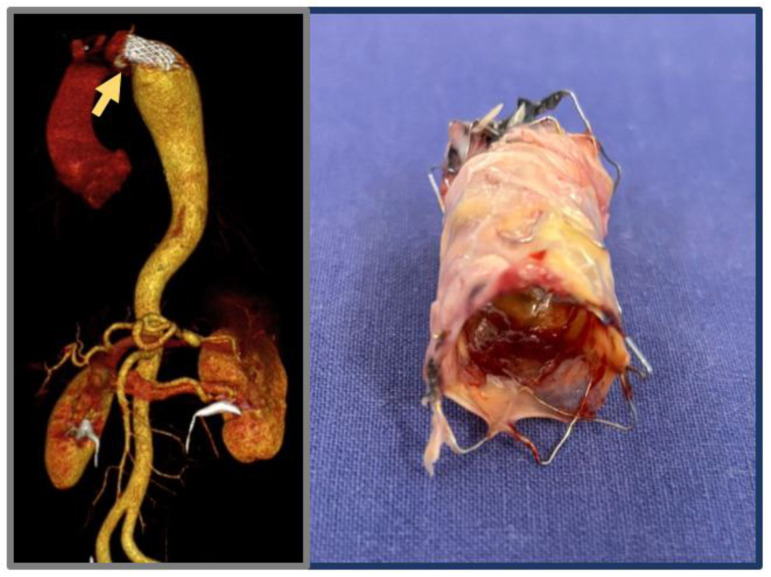
Three-dimensional volume rendering reconstruction of the aneurysmal degeneration in a 38-year-old patient after aortic stenting for CoA; details of explanted covered stent.

The selection of the most appropriate strategy for coarctation repair is determined by patient age, anatomical complexity, and the presence of previous interventions. In neonates and infants with discrete coarctation, end-to-end anastomosis remains the standard approach owing to its technical relative simplicity and durable results. Subclavian flap or extended end-to-end repairs are favored in cases of long-segment narrowing or aortic arch hypoplasia, providing effective relief with acceptable risk. Patch aortoplasty is now largely limited to reoperative settings or complex anatomies. In adolescents and adults, endovascular repair with covered stents has become an alternative, offering good safety, reduced morbidity, and sustained long-term efficacy. However, lifelong surveillance is warranted, as endovascular treatment carries a potential risk of aneurysmal degeneration (Figure 4).

## 3. Late Complications After CoA Repair: Post-Aortic Coarctation Aneurysms (pCoAA)

### 3.1. Epidemiology

Post-aortic coarctation aneurysms (pCoAA) are one of the most relevant late complications, with a lifetime incidence up to 50% [3,29,32,33,34]. The incidence varies substantially with the type of repair technique. Patch aortoplasty, particularly when using synthetic materials like Dacron, demonstrates the highest aneurysm rates, with approximately 40% of patients developing aneurysms post-repair, and roughly 5–25% requiring reintervention [32]. In one series of adult patients treated via patch aortoplasty, 32.8% underwent aneurysm-related reoperations after a mean follow-up of 12.3 years [3]. In contrast, simpler techniques such as end-to-end anastomosis and tube graft interposition yield markedly lower aneurysm rates—around 3% and 6%, respectively [35]. Endovascular approaches (balloon angioplasty, bare-metal stents, and covered stents) are associated with aneurysm risks of 4–12% [21,30,31]. Although covered stents reduce the risk of rupture, they do not completely eliminate the occurrence of aneurysms [21,30,31].

Aneurysms can develop or enlarge decades post-repair, with documented presentation even 20–30 years later. Risk factors include older age at initial repair, persistent hypertension, patch-related mechanical stress, associated bicuspid aortic valve disease, and intrinsic medial wall degeneration [26,27,29,33]. In a recent multicenter study, Melissano et al. [29] reported that patch aortoplasty and subclavian flap repair were the primary interventions mostly associated with aneurysm formation (10% and 65%, respectively). Still, also end-to-end anastomosis, bypass graft use, extra-anatomic approach, and endovascular repair were associated with aneurysmal degeneration (accounting together for 26%).

### 3.2. Pathophysiology and Morphological Spectrum

The pathogenesis of post-aortic coarctation aneurysms is multifactorial. Rigid synthetic patches introduce focal stress at the interface with the native aorta, predisposing the area to pseudoaneurysm or true aneurysm formation [36,37]. Native aortic wall often exhibits medial degeneration—characterized by fibrosis, cystic necrosis, and smooth muscle cell loss—that persists post-repair. When combined with hypertension, these lesions may foster aneurysm development [38].

Morphologically, there are different types of aneurysms. Pseudoaneurysms result from suture dehiscence or patch interface disruptions at patch margins. Fusiform or saccular aneurysms may protrude from patched or adjacent segments, frequently asymptomatic until they reach a critical size or rupture. Rupture contributes to approximately 7% of late PCoAA-related mortalities [29,39,40,41,42]

### 3.3. Surveillance: Imaging and Follow-Up Protocols

Due to the potential for late aneurysm degeneration, lifelong surveillance is imperative.

Computed tomography angiography (CTA) is considered the reference imaging modality for stented or complex anatomy [43]. Magnetic resonance imaging (MRI) may be an alternative for routine monitoring due to its high resolution and absence of ionizing radiation; however, metallic stents may cause image artifacts—new MRI sequences or stent-compatible designs mitigate this issue [43]. Transthoracic echocardiography is unsatisfactory for detecting descending thoracic aneurysms (sensitivity < 25%) and cannot be used alone [39,40]. Surveillance intervals include MRI or CTA at 6–12 months post-surgery after patch aortoplasty, then every 3–5 years lifelong [39,43]. Following stent-based repair, imaging at 3–6 months, then every 3 years, is recommended due to the potential for delayed aneurysm formation [41]. Risk factor modification is essential, including aggressive blood pressure control, periodic evaluation for bicuspid aortic valve disease, and routine screening for cerebral aneurysms [29,41]

### 3.4. Clinical Implications and Indications for Intervention

Aneurysm rupture often occurs without warning and could be fatal, contributing to about 7% of late mortality in pCoAA patients [29]. Growth behavior varies widely—some aneurysms enlarge slowly, while others double in size within a year (e.g., 4.5 cm to 8 cm) [29]. Clinical decisions emphasize growth rate, morphological features, and symptomatic presentation [29]. Generally accepted indications for treatment include a maximum aneurysm diameter > 5.5 cm, growth rate > 0.5 cm/year, saccular or pseudoaneurysm morphology, symptomatology, and proximity to high-stress areas such as patch margins or branch vessels [29,35,43].

### 3.5. Therapeutic Strategies: Open Versus Endovascular Interventions

A recent international, multicenter, retrospective cohort study, published by Melissano et al. [29], assessed early and midterm outcomes following surgical and endovascular treatment of pCoAA. These aneurysms, occurring decades after primary aortic coarctation repair, are particularly associated with synthetic patch aortoplasty [29]. The study period was 2000–2021 and included 74 patients treated across 14 high-volume academic cardiovascular centers, actually the largest series reported in the literature. Patients were diagnosed with pCoAA at a median age of 44 years, with a median period of 33 years after prior CoA repair, highlighting the importance of lifelong imaging. Most aneurysms were saccular (median diameter: 54 mm), and 45% were symptomatic, most commonly with chest or back pain. Of the 74 patients, 28 underwent open surgical repair and 46 had TEVAR. Open repair was technically challenging due to prior scarring and anatomical issues. TEVAR often required supra-aortic revascularization (30% of cases). Notably, 65% of aneurysms followed synthetic patch repairs, now known to increase long-term risk [29].

#### 3.5.1. Open Surgical Repair

**Figure 5:**
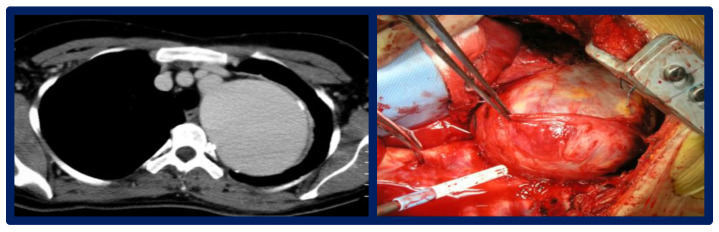
CTA axial view of a giant pCoAA; intraoperative finding.

For complex or anatomically challenging aneurysms, open surgical repair remains the definitive approach. Advantages include direct aneurysm resection and durable graft replacement (Figure 5). However, perioperative morbidity (20–30%) and mortality (3.6%) [29] can be high due to reoperation risks such as bleeding, nerve injury, and pulmonary adhesions in case of re-do thoracic surgery. Paraplegia is an infrequently reported complication in this specific type of thoracic aortic surgery, as the natural collateral circulation associated with aortic coarctation mitigates the risk of spinal cord ischemia [44].

#### 3.5.2. Endovascular Repair (TEVAR)

Thoracic endovascular aneurysm repair (TEVAR) is increasingly favored for suitable patients. Technical success rates approach 98–100%, with perioperative mortality being 2.2% [29]. Benefits include avoidance of thoracotomy in the context of redo operations, reduced blood loss, shorter hospital stays, and avoidance of cross-clamping [29]. Limitations include challenging anatomies such as small access vessels or tortuous aortic arches, and endoleaks reported in approximately 4–15% of cases, requiring regular surveillance [29].

In patients with complex arch involvement, hybrid treatments combining bypass grafting (e.g., carotid-to-subclavian) with TEVAR enable safe coverage of challenging aneurysms. These strategies expand endovascular candidacy but increase operative complexity and invasiveness [29,45].

### 3.6. Comparative Early and Long-Term Outcomes

In a multicenter retrospective cohort of 74 patients, Melissano et al. [29] reported that the in-hospital mortality was low (2.7%), with one death in each group. Major complications included stroke (1.4%), renal failure (2.7%), and vocal cord paralysis (2.7%). No spinal cord ischemia occurred. TEVAR achieved 92.5% technical success. After a median follow-up of 50 months, reintervention was needed in 2.7% of cases. Outcomes were similar between surgical and endovascular approaches [29] (Table 1).

## 4. Late Complications After CoA Repair: Re-Coarctation

### 4.1. Epidemiology

The incidence of re-coarctation varies by age at repair, method of intervention, and anatomical factors. It is more frequent in neonates and infants, with studies reporting re-intervention rates of up to 50% in patients treated in the first month of life [46,47]. In older children and adults, the incidence is significantly lower, ranging from 5% to 20% depending on the technique used [46,47].

Several risk factors for re-coarctation have been identified:

- Young age and low weight at repair: neonates and low-birth-weight infants have smaller vessels, increasing the risk of restenosis [48].
- Aortic arch hypoplasia: associated hypoplasia, especially of the transverse arch, contributes to persistent or progressive gradients post-repair [49].
- Surgical technique: end-to-end anastomosis and subclavian flap repairs have different restenosis rates; the latter is associated with higher re-coarctation risks if not properly sized [49].
- Residual pressure gradients: a persistent gradient >10 mmHg immediately after surgery is a predictor of recurrence [50].
- Genetic syndromes: Turner syndrome, bicuspid aortic valve, and other connective tissue disorders can predispose patients to vascular abnormalities and restenosis.

### 4.2. Pathophysiology and Morphological Spectrum

The mechanism of re-coarctation involves both mechanical and biological processes. Initially, incomplete resection of ductal or fibrous tissue can cause residual narrowing. Over time, neointimal proliferation, scarring, and fibrosis at the site of repair can further reduce luminal diameter. In neonates and infants, the immature vascular matrix and ongoing somatic growth exacerbate this process [51].

Another key factor is arch growth mismatch. In cases where the transverse arch remains hypoplastic postoperatively, blood flow dynamics are altered, causing increased shear stress and promoting restenosis [52]. Additionally, in the case of primary endovascular treatment, systemic inflammation and endothelial injury from balloon angioplasty or stent placement may also contribute to vessel wall remodeling and intimal thickening [52].

### 4.3. Clinical Presentation and Surveillance Imaging

Re-coarctation may present with nonspecific symptoms or be asymptomatic, particularly in young children. Classic clinical findings include: upper-limb hypertension, radiofemoral gradient, systolic murmur over the back or left thorax, and headaches, leg fatigue, or claudication in older children [47,49].

In many cases, re-coarctation is identified through surveillance imaging or abnormal blood pressure measurements rather than symptoms.

Diagnostic modalities include:

- Echocardiography: first-line for evaluating aortic arch gradients and ventricular function. A Doppler gradient >20 mmHg suggests significant obstruction [53]
- MRI/CT angiography: provide detailed anatomical assessment, especially of the transverse arch and collateral vessels
- Cardiac catheterization: gold standard for gradient measurement and anatomical delineation. Peak-to-peak systolic gradient ≥20 mmHg is commonly used to justify intervention [47]

### 4.4. Therapeutic Strategies: Open Versus Endovascular Interventions

Treatment of re-coarctation depends on the patient’s age, anatomy, previous intervention, and severity of the lesion.

#### 4.4.1. Balloon Angioplasty

Balloon angioplasty is the preferred method for infants and small children. It is less invasive than surgery and effective in relieving stenosis. However, there is a 10–30% risk of restenosis, and aneurysm formation is a known complication [54]. Balloon angioplasty is particularly useful in cases of discrete narrowing without arch hypoplasia.

#### 4.4.2. Stent Placement

In older children (>25–30 kg) and adolescents, endovascular stent placement offers a durable solution with low recurrence rates. Covered stents may be used when aneurysm risk is high. Complications include stent fracture, migration, and re-narrowing due to somatic growth [50,54]. Some patients may require stent redilation as they grow.

#### 4.4.3. Surgical Re-Intervention

Surgery is indicated in cases with complex arch anatomy, long-segment narrowing, or failed percutaneous treatment. Surgical options include extended end-to-end anastomosis, patch aortoplasty, or extra-anatomic bypass [47,49]. While surgery carries a higher perioperative risk, it offers definitive repair in select cases (Table 2).

### 4.5. Early and Long-Term Outcomes

Even after successful re-intervention, patients with CoA are at risk for long-term complications. Persistent or recurrent systemic hypertension affects up to 40% of patients, contributing to left ventricular hypertrophy, aortic aneurysms, and premature coronary artery disease.

Lifelong follow-up is recommended, including periodic blood pressure monitoring, imaging, and exercise testing. MRI is preferred for surveillance due to its ability to detect arch abnormalities, stent integrity, and aneurysm formation without ionizing radiation [46,47].

## 5. Conclusions

Post-repair aortic aneurysms remain a major late complication in patients treated for CoA, especially following patch-based repairs. Post-repair aortic aneurysm management should be individualized and centralized in specialized centers, where multidisciplinary teams can optimize treatment strategies based on patient anatomy and comorbidities. Re-coarctation after aortic coarctation repair remains a clinically significant issue, particularly in neonates and infants, but also in adults several years after primary repair. Long-term multidisciplinary follow-up is essential after coarctation repair, focusing on surveillance for cardiovascular and cerebrovascular complications. Persistent hypertension significantly contributes to late morbidity; therefore, periodic cardiovascular evaluation and brain imaging are recommended to ensure early detection of vascular changes and prevention of long-term adverse outcomes. Early recognition through surveillance, accurate diagnosis, and timely intervention are essential for reducing long-term morbidity. While both surgical and catheter-based strategies offer effective treatment, each carries specific risks and benefits given the notable risk of rupture of pCoAA and incidence of recoarctation—even decades post-intervention—lifelong imaging surveillance combined with judicious selection of open, endovascular, or hybrid treatments is crucial.

## Figures and Tables

**Table 1:** Summary of the key points on p-CoAA open and endovascular treatment after CoA repair.

Technique	PROs, CONs	In-Hospital Mortality	Reintervention
Open Surgical Repair	Advantages: direct resection, durable graft. Limitations: technical difficulties related to redo surgery, Risks: bleeding, nerve injury, adhesions; rare paraplegia (collateral circulation protective).	Up to 3.6%	/
Endovascular Repair	Technical success: 98–100%. Advantages: avoids thoracotomy, less blood loss, shorter stay. Limitations: anatomic challenges (tortuous arch, small access vessels), lifelong imaging required. Hybrid (TEVAR + bypass): expands indications but increases complexity	Up to 2.2%	4–15%
Comparative Outcomes	In-hospital mortality: 2.7%. Major complications: stroke 1.4%, renal failure 2.7%, vocal cord paralysis 2.7%. No spinal cord ischemia. Long-term: reintervention 2.7% after ~50 months. TEVAR vs. Surgery: similar midterm outcomes.	Up to 2.7%	/

**Table 2:** Therapeutic Strategies for Re-Coarctation of the Aorta.

Treatment	Indications/Preferred Patients	Advantages	Limitations/ Complications	Long-Term Considerations
Balloon Angioplasty	Infants and small children; discrete narrowing without arch hypoplasia	Minimally invasive, effective for focal stenosis	Restenosis risk up to 30%; aneurysm formation	May require repeat procedures; careful imaging follow-up
Stent Placement	Older children (>25–30 kg), adolescents, adults	Durable lumen expansion; low recurrence; covered stents reduce aneurysm risk	Stent fracture, migration, re-narrowing with growth; need for re-dilation during somatic growth	Lifelong imaging to assess stent integrity; possible re-intervention
Surgical Re-intervention	Complex arch anatomy, long-segment narrowing, failed percutaneous repair	Definitive anatomical repair; multiple surgical options (extended end-to-end anastomosis, patch, bypass)	Technical difficulties related to re-do surgery	Durable in complex cases; reoperation risk in the future if further degeneration occurs

## Data Availability

Data unavailable for consultation due to privacy restrictions.

## Abbreviations

The following abbreviations are used in this manuscript:

CoA	Aortic coarctation
pCoAA	Post-aortic coarctation aneurysm
BAV	Bicuspid aortic valve
CTA	Computerized tomography angiography
MRI	Magnetic Resonance Imaging
OSR	Open surgical repair
TEVAR	Thoracic endovascular aortic repair
